# 2D Digital Image Correlation and Region-Based Convolutional Neural Network in Monitoring and Evaluation of Surface Cracks in Concrete Structural Elements

**DOI:** 10.3390/ma13163527

**Published:** 2020-08-10

**Authors:** Marek Słoński, Marcin Tekieli

**Affiliations:** Faculty of Civil Engineering, Cracow University of Technology, ul. Warszawska 24, 31-155 Kraków, Poland; Marcin.Tekieli@pk.edu.pl

**Keywords:** digital image correlation, region-based convolutional neural network, machine learning, crack monitoring, crack detection and localization

## Abstract

This paper shows how 2D digital image correlation (2D DIC) and region-based convolutional neural network (R-CNN) can be combined for image-based automated monitoring and assessment of surface crack development of concrete structural elements during laboratory quasi-static tests. In the presented approach, the 2D DIC-based monitoring enables estimation of deformation fields on the surface of the concrete element and measurements of crack width. Moreover, the R-CNN model provides unmanned simultaneous detection and localization of multiple cracks in the images. The results show that the automatic monitoring and evaluation of crack development in concrete structural elements is possible with high accuracy and reliability.

## 1. Introduction

Much of the important concrete structures that are in use today were erected several years ago and now they are close to their design life [1]. As a result, the structures require regular condition assessment for understanding of the current state of their structural components. Condition assessment of a concrete structural element involves monitoring of displacements and evaluation of crack development visible on the surface of the element during laboratory mechanical tests. Monitoring is often done using wired contact sensors such as linear-variable-differential transformers (LVDTs). However, these sensors are often difficult to install and maintain.

Crack evaluation typically involves visual inspection by trained staff and simple measuring tools such as a Brinell magnifier. However, such inspection methods can be expensive, dangerous and time-consuming. On the other hand, computer vision methods allow for fully automated extraction of important information from digital images [2,3]. As a consequence, various methods leveraging image-processing techniques and computer vision methods such as digital image correlation and convolutional neural networks have been developed and applied in past decades [4,5,6,7,8,9].

Monitoring and measurement of displacements using computer vision methods are often performed by applying optical flow-based algorithms such as digital image correlation [10]. A detailed review of DIC applications can be found in [7,11]. DIC have been applied for measuring displacements and strains of specimens made of various materials. Mróz et al. [12] presented a feasibility study of DIC in determining strains in concrete exposed to fire.

DIC methods have also been used for assessment of concrete crack development. Helm in [13] shown how to use DIC for assessment of specimens with multiple growing cracks. Similarly, Rui et al. in [14] presented DIC-based measurement system of crack generation and evolution during static testing of concrete sleepers. In [15] Gehri et al. shown a study on an automated crack detection and measurement based on DIC. Finally, DIC techniques allow for measurements and calculations of strains localization and the width of the fracture process zones on the surface of notched concrete beams [16,17].

Crack assessment using DIC methods is very precise but also require huge computational resources and is very time-consuming. As a result, it is mainly used off-line for assessment after the tests. It is also possible to apply other non-destructive monitoring techniques such as Acoustic Emission (AE) [18] or microwave sensors [19]. On the other hand, in recent years, convolutional neural networks (CNN) have been developed and applied for online automatic detection of concrete cracks and structural damage. See for example, recent state-of-the-art reviews [5,6]. In [20] Cha et al. described an autonomous system for structural visual inspection using region-based deep learning for detecting multiple damage types. In [21] a system for real-time crack assessment with wall-climbing unmanned aerial system is presented. Roberts et al. in [22] shown a system for low-cost pavement condition health monitoring and analysis. In [23] Deng et al. presented a region-based CNN with deformable modules for visually classifying concrete crack. Finally, an application of CNN for detection of flaws in concrete using ultrasonic tomography is described in [24].

These two approaches to monitoring and evaluation of surface cracks in concrete structural elements can be combined for better description and assessment of concrete elements. To the best knowledge of the authors of the paper, there is no such a study on combining DIC and CNN algorithms in this context. As a result, this paper is organized as follows. In this first section, the motivation and main goals of the research study undertaken are given. In Section 2, a detailed description of the proposed new method is presented. In Section 3, CNN model development and deployment is outlined. In Section 4, the experimental procedure is described. In Section 5, the results and discussion are given. Finally, in Section 6, the most relevant conclusions are drawn.

## 2. Methodology

**Figure 1:**
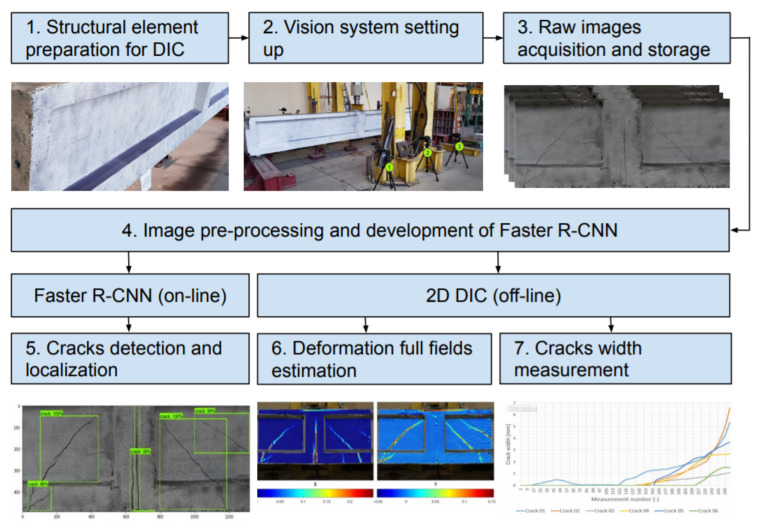
Flowchart for assessment of concrete surface cracks using Faster R-CNN and 2D DIC.

This section provides a description of the proposed methodology. The main goal of the proposed approach is the automatic assessment of the development of concrete cracks by combining two computer vision methods: 2D digital image correlation and region-based convolutional neural network. Figure 1 shows the flowchart of the proposed approach. During the 1st and 2nd step, the tested element is prepared and the vision system is set. In the 3rd step, during the experiment, the digital images are taken and stored using DSLR cameras. In the 4th step, the stored images are pre-processed and the Faster R-CNN model is developed using the stored images. During the 5th step, the developed Faster R-CNN is used for detection and localization of surface cracks on the tested element. In the 6th and 7th step, DIC method is used to compute the displacements, strains and the crack width, respectively. Finally, the automatic assessment of the cracks during the experiments done with Faster R-CNN and DIC can be saved for the later analysis.

### 2.1. Region-Based Convolutional Neural Network

In this paper, to detect and localize multiple cracks on the surface of a tested concrete element a region-based convolutional neural network or regions with CNN features (R-CNN) architecture is applied. The R-CNN model works by performing computations in four steps [25]:

1. a selective search on the input image to select proposed regions containing objects,
2. a pre-trained CNN transforms each proposed region and computes the features extracted from the proposed regions,
3. the extracted features and labeled category of each proposed region are combined to train support vector machine (SVM)-based classifiers for object classification,
4. the extracted features and labeled bounding box of each proposed region are combined to train a linear regression model for bounding box prediction.

**Figure 2:**
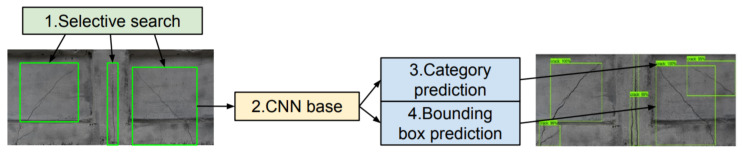
Diagram of the R-CNN model architecture.

This approach was proposed by Girshick et al. in 2013 [26]. The schematic architecture of this model is presented in Figure 2.

**Figure 3:**
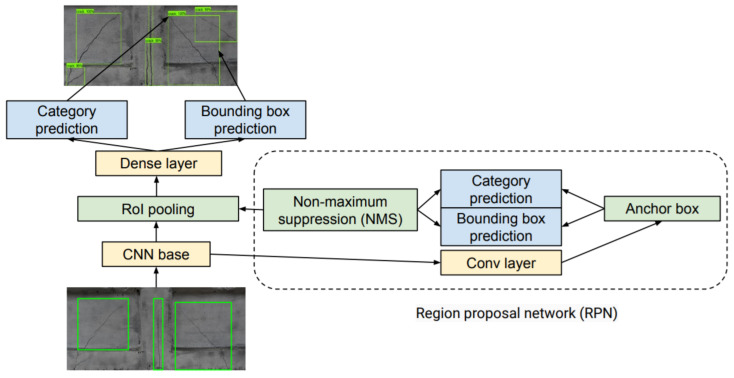
Diagram of the Faster-R-CNN model architecture.

The main bottleneck of the R-CNN model is the need to extract features for each proposed region. The R-CNN model was improved by performing CNN forward computation on the whole image. This improved model is known as a Fast R-CNN model [27]. For obtaining precise object detection, Fast R-CNN requires, in general, many proposed regions in selective search. The Fast R-CNN model was improved by the Faster R-CNN model [28] which replaces selective search with a region proposal network (RPN) and reduces the number of generated proposed regions [25]. The schematic architecture of this model is presented in Figure 3.

The main part of these two architectures is a convolutional neural network (CNN). CNN is a special class of a layered feed-forward artificial neural network. CNN was designed for processing images and audio signals [29]. The typical CNN has an input layer, several hidden layers with nonlinear units, and an output layer with linear units (for regression) or nonlinear units (for classification). Each unit computes a weighted sum of its inputs (activation of the unit). The activation is sent to a transfer function, an S-shaped function such as sigmoid function or rectified linear unit (ReLU) function [29].

The training process use the backpropagation algorithm for efficiently computing the gradient of the loss function and stochastic gradient descent (SGD) algorithm for learning the weights of the CNN model. During training of the convolutional neural network with several millions of parameters, the main issue is the overfitting of the neural model to the dataset. Fortunately, there are several techniques to cope with the overfitting. For example, on can apply transfer learning to a pre-trained model [29]. Transfer learning is a technique used for adaptation of the pre-trained model to another dataset. It is performed by additional training of selected convolutional layers of CNN while the rest of the layers are preserved [29].

In this paper, as a CNN base the Inception V2 architecture is applied. Inception V2 is a convolutional neural network proposed by Szegedy et al. from Google Research in 2016 [30]. These architectures are available through the TensorFlow object detection API [31] which is an open-source library developed by Google Research and built upon TensorFlow [32]. It allows easy development and deployment of CNN-based models for object detection and other computer vision problems.

### 2.2. Digital Image Correlation

Digital Image Correlation is a well-developed and popular tool for evaluation of surface deformations [7,8,10]. It can also be used as a non-destructive method for full-field measurements of displacements of a tested specimen surface. DIC was developed at the University of South Carolina in the early 80s [33,34].

DIC works by processing images taken during the deformation of an object. Then it tries to establish a mapping between the image coordinates of the reference (undeformed) object image and the image coordinates of the deformed object image by searching for the mapping which gives the highest correlation between the reference image and the current image. The mapping is then used for calculating full-field strains [35].

**Figure 4:**
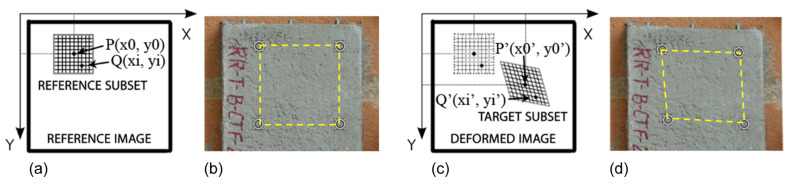
Illustration of the basic principle of the digital image correlation method: sketch of the subset (**a**,**c**) and measurement points on the surface of a specimen (**b**,**d**) before (**a**,**b**) and after (**c**,**d**) deformation [36].

The images are stored as a 2D matrix of pixels and each image is correlated with the reference (undeformed) image. The points of the grid based on the specified image subsets are matched and identified as that associated with the highest value of the correlation coefficient. This coefficient is calculated between the reference subset “f” and the target subset “g”, whose dimensions are equal and are M × N pixels using the zero-mean normalized cross-correlation criterion defined in Equation (1). Illustration of the basic principle of digital image correlation is shown in Figure 4.

(1) $${\mathrm{CC}}^{\mathrm{ZMN}}=\frac{\sum_{i=1}^{M}\sum_{j=1}^{N}\left[\left(f\left(i,j\right)-u_{f}\right)\times (g\left(i,j\right)-u_{g}\right)]}{\sqrt{\sum_{i=1}^{M}\sum_{j=1}^{N}{\left(f\left(i,j\right)-u_{f}\right)}^{2}\times \sum_{i=1}^{M}\sum_{j=1}^{N}{\left(g\left(i,j\right)-u_{g}\right)}^{2}}},$$

where $u_{f}$ is the intensity of the reference subset and $u_{g}$ is the intensity of the target subset form.

## 3. Model-Development Workflow for Crack Detection Based on Faster R-CNN

In this work we have developed a predictive model for crack detection based on Faster R-CNN architecture described in the previous section. The development of the model consisted of several stages. In the first stage we collected and annotated several images of concrete elements with surface cracks. For model development all annotated images were divided randomly into three sets. The first set was used for training, the second for validation images and third for testing the Faster R-CNN model. Finally, the developed model was deployed for crack detection during experiments described in the next section.

### 3.1. Dataset Collection and Annotation

**Figure 5:**
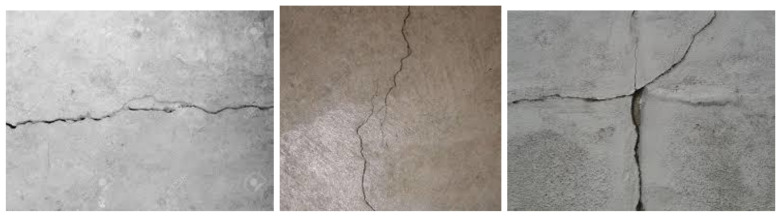
Example of images of concrete elements containing cracks.

Collection and annotation of images for building a concrete crack detector is very important stage because the accuracy of the trained model depends to large extent on the quality of the prepared dataset. For this research, 1058 images containing cracked concrete elements were collected from different laboratory experiments performed at Cracow University of Technology (CUT). Figure 5 shows selected images of concrete element containing cracks.

**Figure 6:**
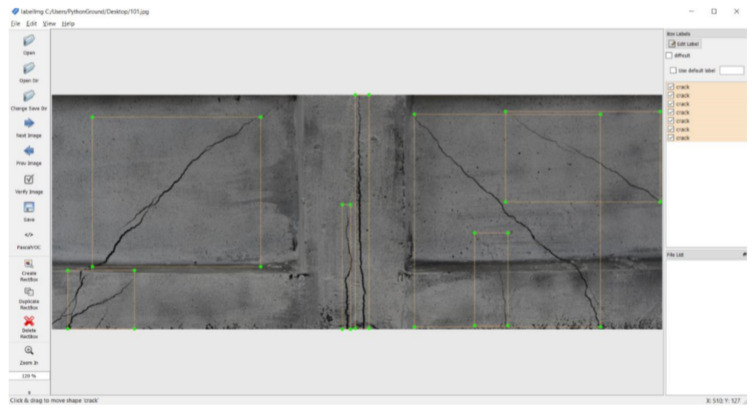
Example of annotation of an image of concrete element containing several cracks.

The images were then manually annotated using a graphical image annotation tool LabelImg [37]. It works by defining a bounding box coordinates and the corresponding label. Figure 6 shows an example of image of concrete element containing several thin cracks.

### 3.2. Model Development

Model development from scratch requires a large number of annotated images. For an object detection task where only small number of training data is available, a common solution is to perform fine-tuning on a CNN which is pre-trained with related source data. In this work, we adopted transfer learning and used a pre-trained convolutional neural network called Inception V2 described in the previous section.

The annotated images were converted to the record format to be used within TensorFlow and the dataset was randomly split in the ratio of 75% for training the model and 25% for testing. This ensures that the model do not overfit to the dataset and would therefore be able to perform well on unseen data.

**Figure 7:**
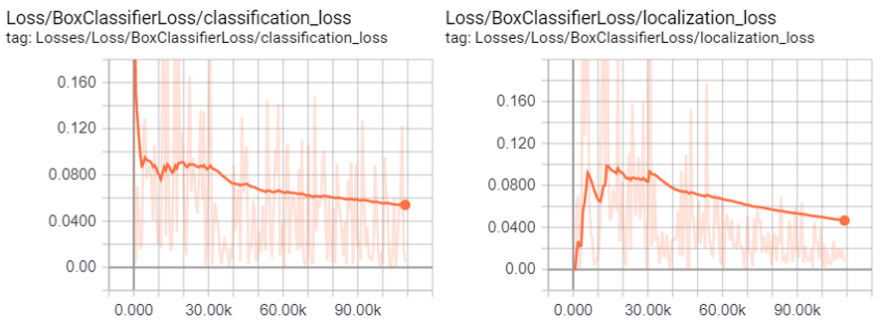
Changes of classification loss and localization loss (with and without smoothing) during the training process (stopped after 11,000 epochs).

The training and validation process of the Faster R-CNN model was performed on a laptop computer with GPU working under the 64-bit Windows 10 operating system using TensorFlow environment and monitored by using TensorBoard system. Figure 7 shows changes of two examples of the monitored losses with and without smoothing (classification loss and localization loss) during the training process. This process was manually stopped after 11,000 epochs which took about 7 h. From the plots, it can be observed that after about 30,000 epochs the smoothed losses started to decrease with constant rates.

**Figure 8:**
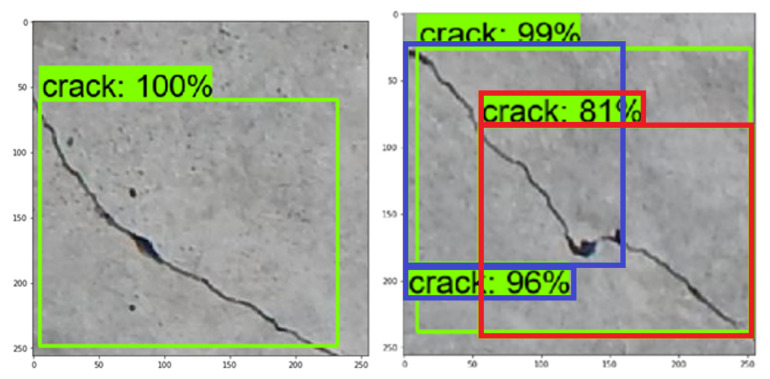
Example of two tested images: the crack on the left image properly detected and localized as one crack (with certainty 100%), the crack on the right image simultaneously properly detected as one crack (with certainty 99%) and not properly detected as two cracks (with certainty 96% and 81%, respectively).

After training, the model was tested by detecting cracks in new images. The model produces a bounding boxes on the images along with a percentage of how accurate this bounding box is based on the trained model. This value provides users with an assessment of how good the detection of cracks is. Figure 8 shows tested images containing cracks. It can be noted that while the crack on the left image was properly detected and localized as one crack with certainty 99% (inside the green bounding box) and also not properly divided into two cracks: inside the blue bounding box (with certainty 96%) and inside the red bounding box (with certainty 81%).

After the model was developed it was deployed for automatic surface crack detection during mechanical tests in laboratory.

## 4. Experiments

In this section, we present the experiments for testing the proposed approach to computer vision-based assessment of surface cracks in concrete structural elements during laboratory experiments. The first part of the assessment consists of monitoring deformation of the element and analyzing development of crack width formed on the surface of the beam to determine the moment in which each of them was created. This part was based on optical measurements and DIC method. In the second part, crack detection and localization using Faster R-CNN model was carried out.

In this work, the proposed methodology is verified by applying it to the laboratory assessment of post-tensioned, precast crane runway beams after more than 50 years of exploitation. They were produced between 1962 and 1963 and were disassembled from the structure at the industrial hall [1]. The three-point bending tests were conducted on girders made of two precast segments, with the total length of the span L = 2 × 290 cm + 20 cm = 600 cm. The girder has an I-section with height H = 80 cm. The segments were connected due to the action of the force and welding of steel sheets to steel marks. The joint was filled by applying fine-grained concrete.

The static three-point bending test yields the maximum bending moment at the joint of the segments. The loading cycle consisted of two stages. In the first stage a relatively small load value was applied and then the beam was unloaded. In the second stage the loading was carried out to the complete collapse of the beam.

**Figure 9:**
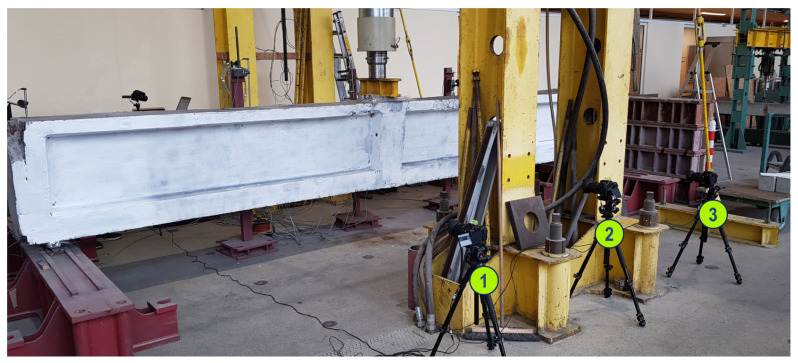
Test stand prepared for monitoring post-tensioned, precast crane runway beam during the three-point bending test using the CivEng Vision system.

The experiments were conducted in the Research Laboratory for Building Materials and Structures at the Cracow University of Technology. The deformation of the beam was monitored by applying optical measurements carried out using 3 synchronized DSLR cameras. The cameras were located at the center of the beam. Before experiments, the side surface of the beam was prepared for vision-based measurements by adding random distribution of black dots with spray paint on whitewashed surface of the beam. Figure 9 shows the test stand prepared for this research with the numbering of acquisition devices.

**Figure 10:**
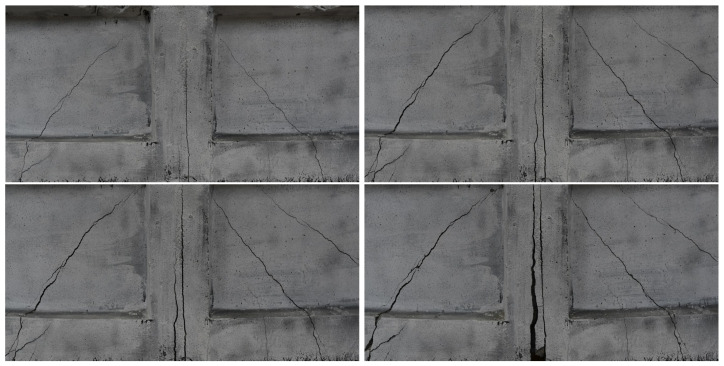
Images of the side surface of the beam, taken using the CivEng Vision system, during the three-point bending test.

The CivEng Vision system, developed at Cracow University of Technology (CUT), was used for acquisition, storing and processing the images [38,39]. The images were then processed using DIC method for computing deformation fields and crack width visible on the surface of the beam. Finally, the images were processed by trained R-CNN model to automatically detect and localize the cracks on the surface. Figure 10 shows four images of the side surface of the beam, taken using the CivEng Vision system, during the three-point bending test.

**Figure 11:**
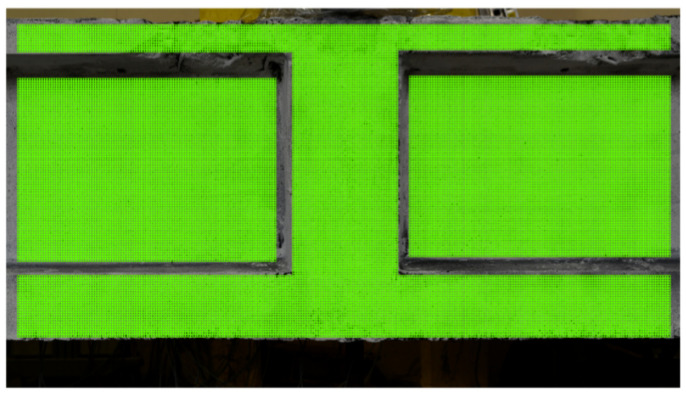
Grid of subsets placed on the surface of the tested crane runway beam.

Figure 11 shows grid of subsets for monitoring deformation of the surface of the tested crane runway beam using DIC.

## 5. Results and Discussion

In this section, we present the results of application of digital image correlation and region-based convolutional neural networks in evaluation of surface cracks in the crane runway beam during experiments described in the previous section.

### 5.1. Monitoring of Beam Side Surface Deformation Fields

**Figure 12:**
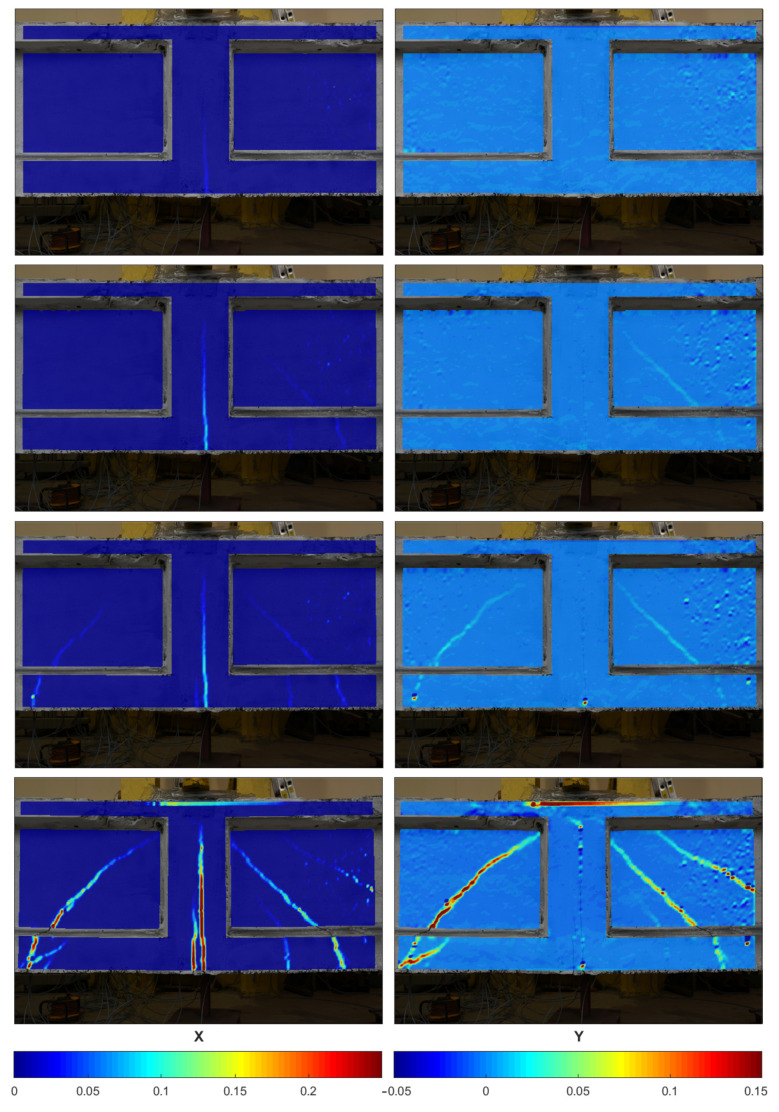
Deformation fields on the surface of the beam in X and Y direction during loading phases measured by DIC.

The deformation fields of the beam side surface was monitored using DIC method. Figure 12 shows the changes of deformation fields in X and Y direction during the loading of the beam. As can be seen in the first row and the first column, the first vertical crack formed exactly in the middle of the beam span (opening of the joint) in the first phase of the loading and was observed under the load P = 287 kN. It is worth mentioning that no cracks were observed, during the initial phase of the loading, by visual inspection of the surface.

The next two rows present the development of vertical and diagonal cracks during the experiments. The first diagonal crack was observed using DIC after applying loading P = 454.8 kN in the second phase. The last row shows the deformation fields just before the failure. The element was damaged at the load of P = 837.7 kN. Failure of the element was signalized through the opening of the joint and by occurring diagonal cracks. In the final phase of beams operation, perpendicular cracks have occurred connecting with diagonal cracks and a large increase of deflection was observed. The girder was finally damaged at the joint as a result of significant strains in prestressing cables.

It is worth adding that deflection measured by the DIC was very close to the same as the deflection indicated by LVDT device. The difference was below 1 mm.

### 5.2. Assessment of Crack Width

**Figure 13:**
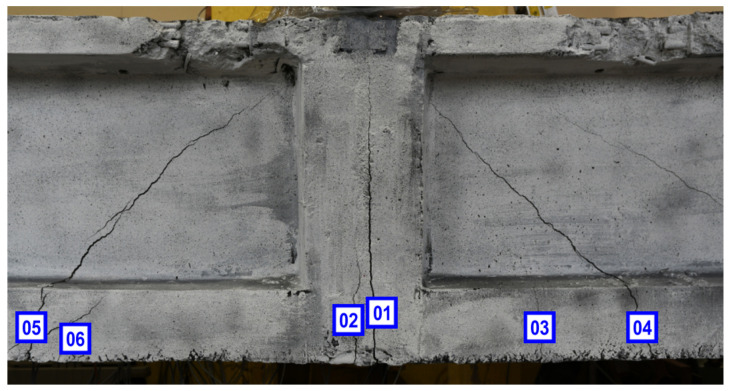
Numbering of the analyzed cracks [40].

Optical measurements and DIC were also used to analyze the development of cracks visible on the surface of the beam and to measure their width. It is also possible to determine the moment the crack starts to develop. In Figure 13 shows the numbering of the analyzed cracks.

**Figure 14:**
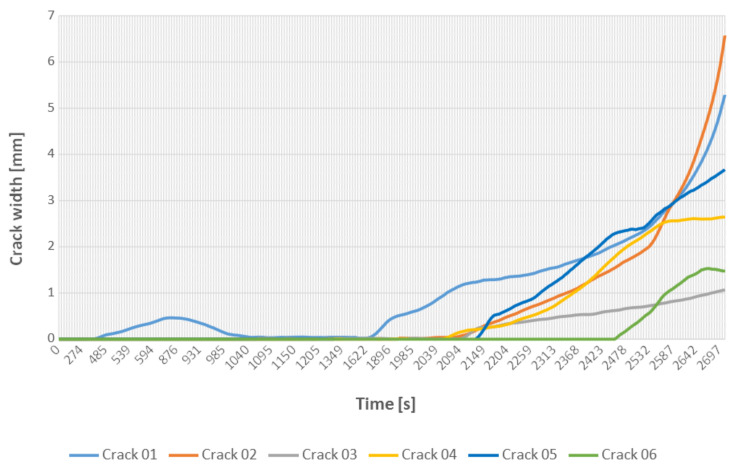
The crack width development during the loading phases.

As can be seen in Figure 14, the main vertical crack (Crack 01) formed exactly in the middle of the beam span and appeared in the first phase of the loading. Then the load was reduced to zero and the crack was almost completely closed. It appeared again during the second phase of the experiment.

### 5.3. Cracks Detection and Localization

To provide an assessment of the trained Faster R-CNN for detecting and localizing concrete cracks, new images of cracked concrete elements taken during laboratory experiments on prestressed crane runway beam in the three-point bending test were provided to the trained model.

**Figure 15:**
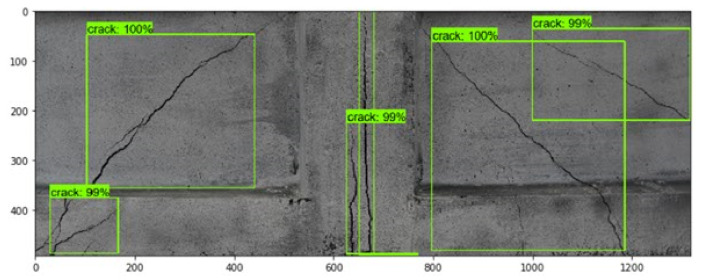
Detected and localized surface cracks (green rectangles) in the testing image of concrete beam using trained Faster R-CNN model.

Figure 15 shows detected and localized cracks in concrete beam with the Faster R-CNN. As can be seen, the network correctly detected and localized all the cracks (diagonal and vertical) visible in the image (green rectangles) with high certainty (more than 98%).

## 6. Summary and Final Conclusions

In this paper, we have developed a computer vision system for automatic assessment of crack development visible on the surface of a concrete structural element during laboratory quasi-static tests. This approach combines 2D digital image correlation for monitoring the development of crack width and trained region-based convolutional neural network for automated detection and localization of multiple cracks. The intention of this work was to provide researchers and engineers with a description of easy-to-use computer vision-based system for quick assessment not only the crack width that are visible on the surface of the tested concrete element but also an automatic approach to crack detection and localization for monitoring purposes.

The computer vision system was evaluated during static tests performed in the CUT laboratory, to set up a system capable of carrying out this task. The images were captured using three DSLR cameras connected by one automatic trigger. The system showed high accuracy in assessment of the surface cracks. The system has the ability to automatic identify the cracks number and their localization. This process can be repeated for static tests of other concrete elements. Once the system was created it can be deployed for use by researchers and engineers for the concrete crack development analysis.
